# Dynamic equilibrium on DNA defines transcriptional regulation of a multidrug binding transcriptional repressor, LmrR

**DOI:** 10.1038/s41598-017-00257-x

**Published:** 2017-03-21

**Authors:** Koh Takeuchi, Misaki Imai, Ichio Shimada

**Affiliations:** 1 Biomedicinal Information Research Center & Molecular Profiling Research Center for Drug Discovery, National Institute of Advanced Industrial Science and Technology, Aomi 2-3-26, Koto-ku, Tokyo 135-0064 Japan; 2 PRESTO, JST, Aomi 2-3-26, Koto-ku, Tokyo 135-0064 Japan; 3 Research and Development Department, Japan Biological Informatics Consortium, Aomi 2-3-26, Koto-ku, Tokyo 135-0064 Japan; 4 Graduate School of Pharmaceutical Sciences, The University of Tokyo, 7-3-1 Hongo, Bunkyo-ku, Tokyo 113-0033 Japan

## Abstract

LmrR is a multidrug binding transcriptional repressor that controls the expression of a major multidrug transporter, LmrCD, in *Lactococcus lactis*. Promiscuous compound ligations reduce the affinity of LmrR for the *lmrCD* operator by several fold to release the transcriptional repression; however, the affinity reduction is orders of magnitude smaller than that of typical transcriptional repressors. Here, we found that the transcriptional regulation of LmrR is achieved through an equilibrium between the operator-bound and non-specific DNA-adsorption states *in vivo*. The effective dissociation constant of LmrR for the *lmrCD* operator under the equilibrium is close to the endogenous concentration of LmrR, which allows a substantial reduction of LmrR occupancy upon compound ligations. Therefore, LmrR represents a dynamic type of transcriptional regulation of prokaryotic multidrug resistance systems, where the small affinity reduction induced by compounds is coupled to the functional relocalization of the repressor on the genomic DNA via nonspecific DNA adsorption.

## Introduction

The excretion of toxic compounds is essential to maintain cellular survival. Therefore, multidrug resistance (MDR) systems are ubiquitously distributed in all three kingdoms of life. MDR phenotypes are often associated with the increased membrane expression of multidrug transporters that excrete toxic compounds^1–3^. High-level expression of multidrug transporters is a major threat in the treatment of infectious diseases with antibiotics and, in human cancer, it reduces the curative effects of medicines against cancer^4, 5^.

The expression of multidrug transporters is regulated by multidrug binding transcriptional regulators^6^, which have the ability to bind structurally diverse toxic compounds that are often the same or overlapping with those excreted by their respective multidrug transporters^7^. Therefore, the multidrug binding transcriptional regulators are the sensors in the MDR systems, enabling the cells to efficiently increase the expression of the required multidrug transporters in response to the toxic compounds.

**Figure 1:**
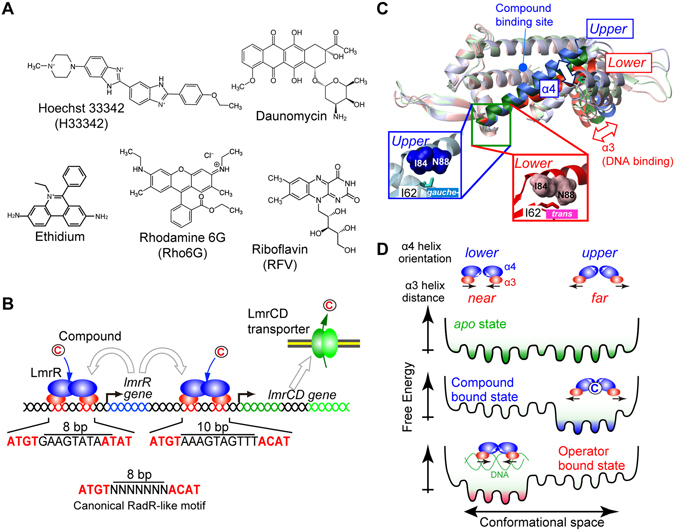
Ligands, regulatory mechanism, and structural properties of LmrR. (**A**) Chemical structures of compounds that are able to bind to LmrR. (**B**) Schematic representation of gene regulation by LmrR. LmrR regulates the transcription of both the *lmrR* and *lmrCD* genes by binding to their respective operator regions. White arrows indicate the destinations of proteins that are translated from each gene. (**C**) Different orientations of the C-terminal α4 helix. A superposition of the LmrR dimer structures is shown (3F8C, blue; 3F8B, green; 3F8F, red). Inset: close-up view of the hinge region. The Ile62 χ^2^ angle is defined by the α4 helix orientation. The α4 helix orientations are coupled to the distance between the α3 helices. (**D**) Schematic representation of the LmrR conformational equilibrium in the *apo*, compound-bound, and DNA-bound states, which underlies the reciprocal compound/operator binding by LmrR.

In the Gram-positive bacterium *Lactococcus lactis*, the MDR activity towards a set of structurally unrelated toxic compounds, such as Hoechst 33342 (H33342), daunomycin, ethidium, and rhodamine 6G (Rho6G)^8, 9^, is achieved through a heterodimeric multidrug transporter, LmrCD^10^ (Fig. 1A). In the *L*. *lactis* genome, the two genes that encode the LmrC and LmrD proteins are adjacent to each other in the same direction, and their transcription is initiated from the shared promoter (hereafter, we refer to them as the *lmrCD* genes). It was demonstrated that the *lmrCD* genes are constitutively expressed in *L*. *lactis*, and that the exposure to toxic compounds elevates the expression of the *lmrCD* genes by up to two-fold for cell survival^11, 12^. Since the knockout of the *lmrCD* genes makes *L*. *lactis* more susceptive to the toxic compounds at concentrations lower than the inducible concentration^10^, the basal constitutive expression of the transporter also plays an important role in the fundamental defense against the toxic compounds.

The basal and induced expression of the *lmrCD* genes is regulated by a multidrug binding transcriptional repressor, LmrR, a homodimeric transcriptional repressor that belongs to the PadR-like family^7, 13^. LmrR is encoded in the same cluster as the *lmrCD* genes^12^ (Fig. 1B). In the absence of toxic compounds, LmrR occupies the promoter/operator region of the *lmrCD* genes and its own gene (*lmrR*), repressing their transcription to the basal level (Fig. 1B). DNA foot-print studies identified imperfect inverted repeats, similar to the PadR-consensus sequences (ATGT (X)_n_ ACAT), in the operator region of the genes^11, 14^, which presumably represent the specific LmrR binding sites (Fig. 1B). In the canonical motif, the two half-sites in the PadR-consensus sequence are separated by 8 base pairs (bp); however, they are separated by 10 bp in the *lmrCD* operator^11^.

Structural studies revealed that the LmrR dimer possesses a hydrophobic pore formed by the α1 and α4 helices at its center to accommodate the compounds (Supplementary Fig. S1)^15, 16^. In the previous study, we demonstrated that LmrR exists as a conformational ensemble with multiple α4 helix orientations in solution (Fig. 1C and D)^17^. While the *upper* and *lower* α4 helix conformations are almost equally present in the *apo* state, the compound ligation to LmrR shifts the conformational ensemble to a higher proportion of the *upper* α4 helix orientations (Fig. 1D)^17^. The structure of the LmrR-DNA complex has not been determined; however, our study has shown that the binding to the *lmrCD* operator shifts the conformational ensemble of LmrR to the *lower* α4 helix orientations, which is opposite to that for the compound ligations^17^ (Fig. 1D). Since the α4 helix orientations are coupled to the relative distance between the DNA-binding α3 helices in the LmrR dimer (Fig. 1C), the distinct conformational ensemble induced by a compound and DNA is assumed to represent the structural basis for the transcriptional regulation by LmrR^17^. This observation strongly suggests that multidrug recognition as well as the transcription regulation of LmrR is described in the conformational selection model for protein interactions^18^.

Although the correlation between the α4 helix conformational ensembles and the binding affinity to the PadR-consensus sequences remains to be investigated, the compound ligation has been shown to reduce the affinity of LmrR for a long stretch (~1000 bp) of the *lmrR* or *lmrCD* promoter/operator regions by several fold^11, 16^. The reduction of affinity upon compound ligation, however, is orders of magnitude smaller, as compared to those of other transcriptional repressors that are controlled by specific inducers. For example, the *lac* repressor reduces the affinity to the operator by ~1000-fold upon binding to its specific inducers^19^. The conformational equilibrium of LmrR is reportedly not fully suppressed even in the compound-bound state and is required for promiscuous multidrug interactions^17^. The small reduction of the operator binding affinity upon compound ligation might arise from this dynamic nature of the LmrR-compound complexes, and thus a different molecular mechanism should underlie to effective connection of the small affinity reduction to the functional release of the transcriptional repression *in vivo*.

In this study, we conducted biochemical and structural analyses of LmrR in complex with the *lmrCD* operator as well as the genomic DNA fragments of *E*. *coli*, which does not contain the specific interaction sequence for LmrR. We found that two LmrR dimers bind to one *lmrCD* operator, which is uncommon for prokaryotic transcriptional repressors. In addition, the relatively high endogenous LmrR concentration (3.2 μM) and the mid μM non-specific DNA binding affinity of LmrR were revealed by biochemical analyses, which implied that LmrR is always bound to the genomic DNA *in vivo*. In this case, the effective affinity to the *lmrCD* operator would be defined by the equilibrium between the operator-bound and non-specific DNA adsorption states. The effective *K*_D_ value of LmrR for the *lmrCD* operator at equilibrium is close to its intracellular concentration, presumably due to the autonomous regulation of its own transcription. These features maximize the effects of the affinity reduction caused by the promiscuous compound ligations, together with the 2:1 binding stoichiometry, and explain both the basal and compound-induced expression of the *lmrCD* gene *in vivo*. The non-specific DNA adsorption model provided here represents a model for the transcriptional regulation in multidrug resistance systems, where the shift in the conformational ensembles induced by promiscuous compound ligations is effectively coupled to the dynamic relocalization of the transcriptional repressors in the genomic DNA through an equilibrium between the operator-bound and non-specific DNA-adsorption states.

## Results

### Binding stoichiometry of LmrR to the PadR-consensus sequence of the *lmrCD* operator

**Figure 2:**
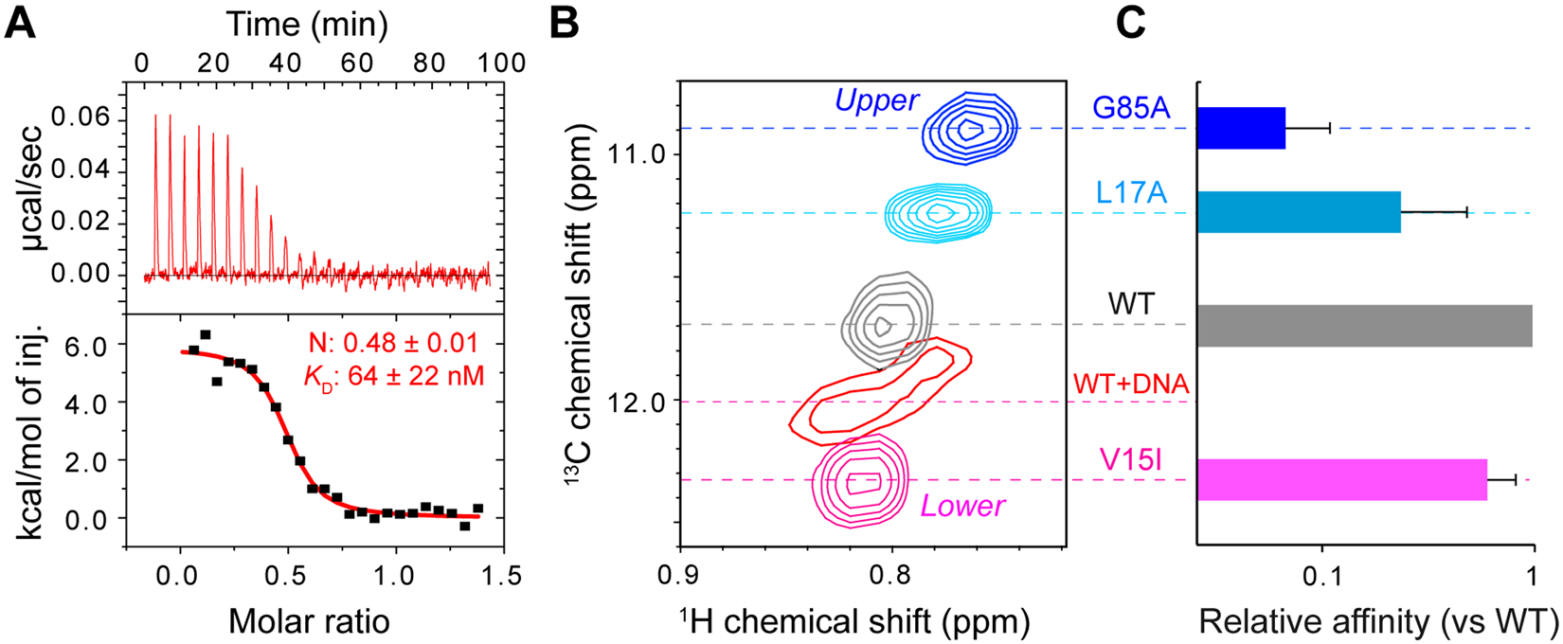
Interaction between LmrR and the PadR-consensus sequence of the *lmrCD* operator. (**A**) ITC measurements of the interaction between LmrR and the *lmrCD* operator. The *lmrCD* operator was titrated to LmrR. (**B**) The Ile^62 1^H_δ1_-^13^C_δ1_ signal of wild-type LmrR and the conformationally biased LmrR mutants. The wild-type LmrR signal in complex with the *lmrCD* operator is also shown. (**C**) DNA affinities of the conformationally biased LmrR mutants relative to the wild-type LmrR. Data are representative of two independent experiments.

To characterize the binding of LmrR to the PadR-consensus sequence of the *lmrCD* operator, we performed isothermal titration calorimetry (ITC) measurements. Previous reports suggested that LmrR interacts with the promoter/operator region of the *lmrCD* genes, including the PadR-consensus sequence in the operator site^11, 14^. Therefore, we used a 33-bp DNA fragment that contains the PadR-consensus sequence of the *lmrCD* operator (hereafter, the *lmrCD* operator) for the interaction analyses^17^. The previous surface plasmon resonance (SPR) analyses indicated that LmrR has a low μM affinity to the *lmrCD* operator^17^. However, the exact stoichiometry of the binding has not been determined. Titration of the *lmrCD* operator to LmrR indicated that the interaction is endothermic, and the *K*_D_ value of the interaction was 64 ± 22 nM (Fig. 2A). Since the affinity of LmrR to the *lmrCD* operator is similar to that reported for the longer *lmrCD* promoter/operator region^11, 16^, this result implies that the PadR-consensus sequence represents a major LmrR binding site in the *lmrCD* promoter/operator region.

The ITC results also showed a 2:1 binding stoichiometry, in which two LmrR dimers bind to one *lmrCD* operator with an equilibrant affinity (Fig. 2A). This stoichiometry was rather unexpected for prokaryotic transcription repressors; however, it was confirmed by the opposite titration, in which LmrR was titrated to the *lmrCD* operator (Supplementary Fig. S2). In addition, SEC analyses indicated the presence of the 2:1 complex in solution and the apparent molecular weight of the fraction closely matches with the expected molecular weight of the 2:1 LmrR dimer: *lmrCD* operator complex (78 K; Supplementary Fig. S2). The NMR titration experiment also confirmed that LmrR is fully in bound from with the *lmrCD* operator at 2:1 stoichiometric concentration and the chemical shift changes induced by the *lmrCD* operator was saturated at the 2:1 stoichiometry (Supplementary Fig. S2). Furthermore, the analytical ultracentrifugation sedimentation velocity experiments of a 2:1 LmrR dimer:*lmrCD* operator solution showed a peak sedimentation values of 7.26S with an estimated molecular weights of 87.8 K (Supplementary Fig. S2), which is closely matched with the molecular weight of the 2:1 LmrR dimer-*lmrCD* operator complex (80.8 K). Therefore, we concluded that two LmrR dimers bind to one PadR-consensus sequence in the *lmrCD* operator.

### The α4 helix conformational ensemble defines the LmrCD operator binding affinity

Our previous study showed that the binding to the compounds and the *lmrCD* operator induces an opposite shift in the α4 helix conformational ensembles (Fig. 1E)^17^. Since the orientations of the α4 helix are coupled to the relative distances between the DNA-binding α3 helices, the shift in the α4 helix conformational ensembles by the promiscuous compound ligations would reduce the population of LmrR that adopts the distance between the α3 helices suitable for the operator DNA binding. The incompatible conformational ensembles would represent the structural basis of the reciprocal compound/operator interaction. However, a direct correlation between the shift in the α4 helix conformational equilibrium and the binding affinity of LmrR for the PadR-consensus sequences has not been investigated.

To address this issue, we selected three mutants (V15I, L17A, and G85A)^17, 20^ and tested their binding affinity to the *lmrCD* operator. These mutations are substantially shifting the conformational equilibrium of the α4 helix from that of wild-type, although the mutation sites are located outside the putative DNA-binding interfaces in LmrR. Therefore, the changes in DNA binding affinity would be due to allosteric shifts in the conformational equilibrium but not by the direct effect of mutations. The overall dispersion of mutant-derived NMR signals are similar to those from wild-type, indicating that no major structural destruction such as monomerization and aggregation is associated with the mutations (Supplementary Fig. S3), which is also supported by the SEC analysis of the mutants (Supplementary Fig. S3).

The changes in the α4 helix conformational equilibrium were confirmed by the ^13^C chemical shift of the Ile^62^ δ1 resonances, which reports the proportion of the *upper* and *lower* α4-helix orientations in each state (Fig. 2B)^20^. The G85A mutant showed the most substantial high-field chemical shift change, which indicates the highest proportion of the *upper* α4 helix orientations among these three mutants. The L17A mutant also showed an increased proportion of the *upper* α4 helix orientations relative to wild-type LmrR. In contrast, the V15I mutant showed a conformational shift to the lowest proportion of the *upper* α4 helix orientations, which is even smaller than that of LmrR bound to the *lmrCD* operator. The *upper* biased mutants exhibited a significant reduction in their affinity to the *lmrCD* operator (Fig. 2C). The reduction in the affinity correlates with the discrepancy in the α4 helix conformational ensemble, relative to those of the wild-type LmrR bound to the *lmrCD* operator. The results represent the direct link between the shift in the α4 helix conformational ensemble and the decrease in the LmrR affinity for the *lmrCD* operator.

### Reduction of the *lmrCD* operator affinity by the compound-bound ligation

**Figure 3:**
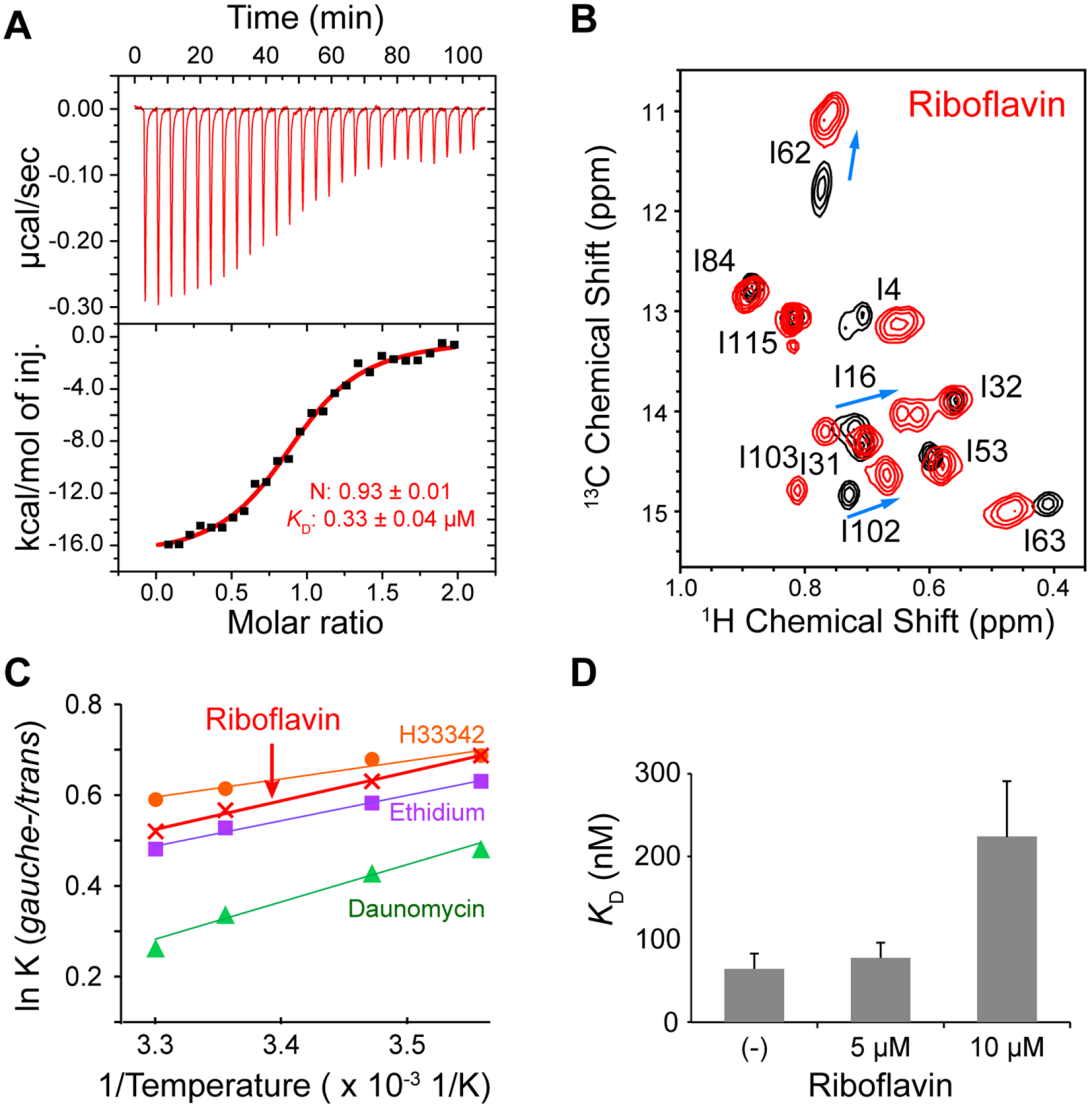
Effect of riboflavin ligation on the LmrR conformation and the LmrR-DNA interaction. (**A**) ITC measurements of the interaction between LmrR and riboflavin. Riboflavin was titrated against LmrR. (**B**) The Ile ^1^H_δ1_-^13^C_δ1_ resonances of LmrR in the *apo* state (black) were overlaid with the riboflavin-bound state resonances (red). (**C**) van’t Hoff plot for the Ile^62^ χ^2^ angle rotameric equilibrium in the compound-bound states. The Ile^62^ δ1 ^13^C chemical shifts were used to calculate the population of each conformer, assuming the exchange between the *gauche-* and *trans* rotameric states^20^. (**D**) Riboflavin ligation to LmrR reduces its binding affinity to the *lmrCD* operator. Data are representative of two independent experiments.

The structure of LmrR in complex with riboflavin was determined by X-ray crystallography^16^. Unlike other compounds that bind to LmrR, the affinity of riboflavin to DNA is weak, in the sub mM range^16^. Therefore, riboflavin preferentially binds to LmrR in the LmrR/DNA mixture and the use of riboflavin is advantageous for analyzing the effect of the compound binding on the interaction between LmrR and the operator. The ITC measurement demonstrated the 1:1 binding stoichiometry, and the *K*_D_ value for the LmrR-riboflavin interaction was 0.33 μM (Fig. 3A), which is consistent with the previous study^16^.

In order to determine whether riboflavin binding induces a conformation change in LmrR similar to that elicited by other compounds, an NMR titration experiment was performed. The NMR experiment revealed that riboflavin binding to LmrR induces a high-field shift of the Ile^62^ δ1 resonance in the ^13^C dimension (Fig. 3B). Thus, similar to other compounds, riboflavin binding shifts the conformation ensemble to a higher proportion of the *upper* α4 helix orientations in LmrR. In addition, the van’t Hoff plot of the Ile^62 13^C_δ1_ chemical shift in the riboflavin-bound state showed a temperature dependence similar to those of other compound-bound states (Fig. 3C). Therefore, the compounds share common structural and energetic properties in the bound states.

Riboflavin binding reportedly reduced the affinity of LmrR to the *lmrCD* promoter/operator region by several fold^16^; however, the experiment was performed with a long stretch (~1000 bp) of the *lmrCD* promoter/operator site. Therefore, we tested the effects of riboflavin binding on the LmrR interaction with the shorter *lmrCD* operator. The addition of riboflavin caused a concentration-dependent decrease in the DNA binding affinity (Fig. 3D). A 3.4-fold reduction of the LmrR affinity to the *lmrCD* operator sequence was observed with 10 μM riboflavin (from 64 nM to 220 nM, Figs 2A and 3D), without any change in the binding stoichiometry (Supplementary Fig. S4). The results confirmed that the riboflavin binding reduces the LmrR affinity to the *lmrCD* operator by several fold. Given the shared structural and energetic properties of the compound-bound states, a similar affinity reduction would be expected for other compounds upon the binding to LmrR.

### Transcriptional regulation of the *lmrCD* genes by LmrR *in vivo*

**Figure 4:**
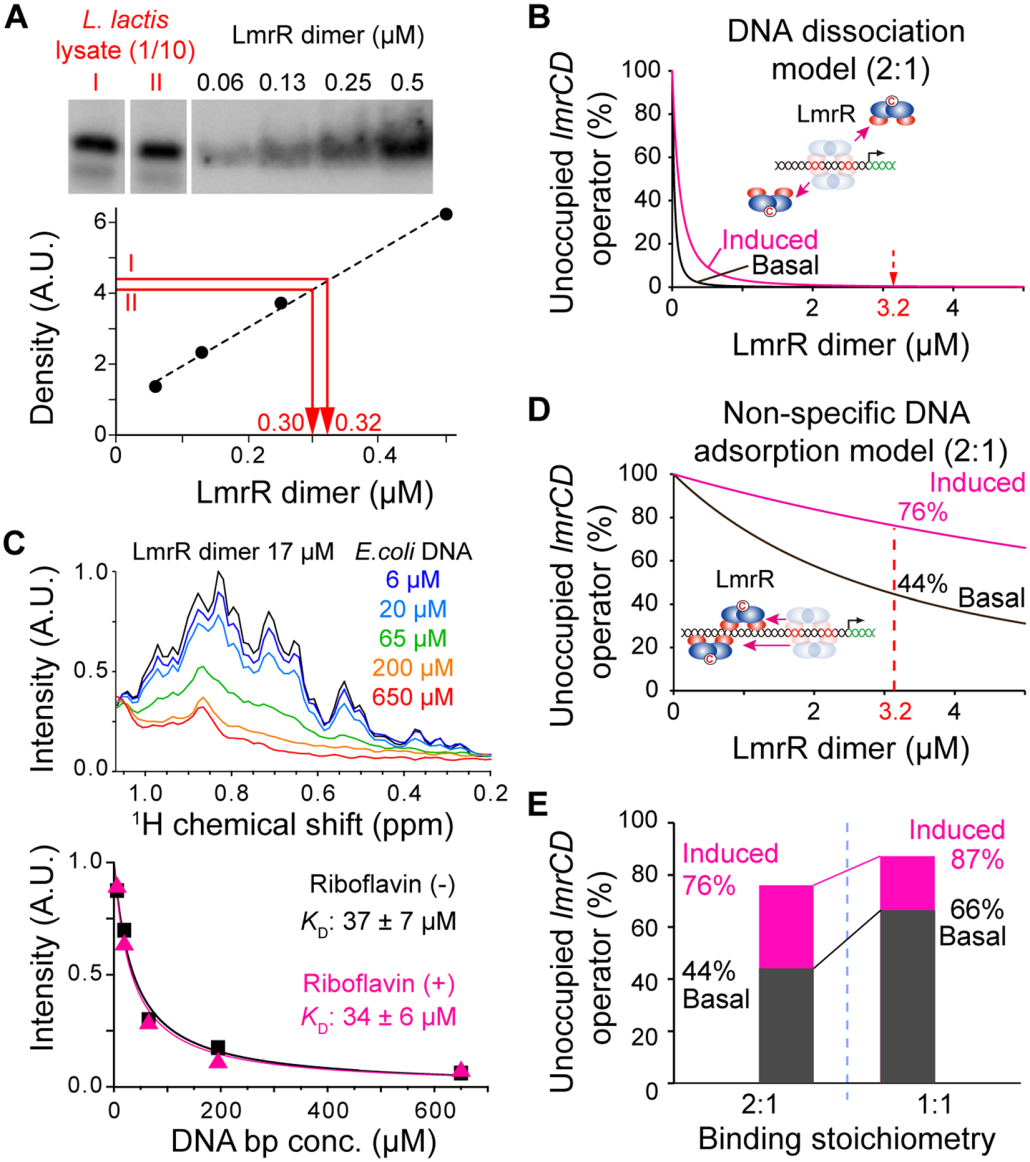
Transcriptional regulation of LmrR. (**A**) Western blot analysis of the intracellular LmrR concentration. The figure is originating from a single image but juxtaposed for clarification as indicated by the white bars. (**B**) Population of the unoccupied *LmrCD* operator in the DNA dissociation model. Black and magenta graphs represent the populations of the unoccupied *lmrCD* operator without and with riboflavin, respectively. (**C**) Determination of non-specific DNA absorbance, using the titration of the *E*. *coli* genomic DNA fragments against LmrR. Black and magenta graphs in the lower panel represent the intensities of LmrR resonances without and with riboflavin, respectively. (**D**) Population of the unoccupied *lmrCD* operator in the non-specific DNA adsorption model. Color codes are the same as in (**B**). (**E**) Population change of the unoccupied *lmrCD* operator in the non-specific DNA adsorption model with 2:1 (left) and 1:1 (right) binding stoichiometries. Color codes are the same as in (**B**). Data are representative of two independent experiments.

The previous biochemical analyses^16^, as well as the current ITC experiments, indicated that the reduction of the LmrR affinity to the *lmrCD* operator upon the compound ligations is relatively small (less than 10-fold). To determine whether the affinity reduction is sufficient to evoke the LmrR dissociation from the *lmrCD* operator and the subsequent induction of the *lmrCD* gene expression, the population of the *lmrCD* operator that is not occupied by LmrR should be estimated from the binding affinity and concentrations. For this purpose, the endogenous concentration of LmrR was determined, using an antibody against LmrR (Fig. 4A). Western blot analyses revealed that the endogenous concentration of LmrR is 3.2 ± 0.2 μM. The concentration of the single *lmrCD* operator present in the *L*. *lactis* genome was estimated to be 1.5 nM, assuming the 1.2 μm × 1.5 μm ellipsoid shape and 1.1 × 10^−15^ L volume of *L*. *lactis* cells.

If a simple equilibrium between the free state and the *lmrCD* operator bound state is assumed for the transcriptional regulation (hereafter we call this model as the DNA dissociation model)^6^, then the number of *lmrCD* operators that are not occupied by LmrR is almost zero in both the normal and riboflavin-bound states (Fig. 4B). Therefore, the transcriptional regulation by LmrR cannot be achieved by the DNA dissociation model, and a different mechanism should be considered.

Given the 2.4 M bp genome size, the estimated base pair concentration of DNA in *L*. *lactis* cells is 3.6 mM. This implies that the contribution of non-specific DNA adsorption to the genomic DNA might not be negligible^21^. In order to estimate the extent of the non-specific DNA adsorption, sonicated *E*. *coli* genomic DNA was titrated against LmrR. The *E*. *coli* genomic DNA comprises 4.6 M bp and lacks a sequence identical to the PadR-consensus sequence of the *lmrCD* operator. The sonicated *E*. *coli* genomic DNA consisted of DNA fragments ranging from 100 to 2000 bp with an average length of ~500 bp. Since the size each *E*. *coli* genomic fragments is larger than the length of LmrR dimer, which corresponds to 22 bp, multiple binding sites with different affinity can be anticipated for each fragment. Therefore, we determined the apparent affinity par base pair for the nonspecific interaction between LmrR and the *E*. *coli* genomic fragments. The affinity of LmrR to the non-specific DNA fragments was determined by NMR titration experiments (Fig. 4C; upper panel), and the apparent *K*_D_ value was 37 ± 7 μM in the *apo* state (Fig. 4C, lower panel, black). This suggests that the LmrR in *L*. *lactis* cells is fully adsorbed on the genomic DNA *in vivo*. The affinity to the non-specific genomic DNA fragments did not change by the addition of 25 μM riboflavin (Fig. 4C, lower panel, magenta), presumably because the non-specific DNA adsorption does not induce conformations that are incompatible for the riboflavin binding. Therefore, the equilibrium between the *lmrCD* operator-bound and non-specific DNA adsorption states in the *L*. *lactis* genome seems to define the occupancy of LmrR at the *lmrCD* operator (hereafter, we call this model the non-specific DNA adsorption model).

The effective affinity (*K*_D_^eff^) to the operator, in the non-specific DNA adsorption model, can be described as follows^21^:

1 $${K}_{{\rm{D}}}^{eff}={K}_{{\rm{D}}}^{{\rm{S}}}(1+{C}_{{\rm{A}}}/{K}_{{\rm{D}}}^{{\rm{A}}}),$$

where *K*_D_^S^ and *K*_D_^A^ represent the *K*_D_ and apparent *K*_D_ values to the specific operator and the non-specific adsorptive sites, respectively, and *C*_A_ is the concentration of the non-specific adsorptive sites (3.6 mM). According to the equation, the *K*_D_^eff^ of LmrR for the *lmrCD* operator in *L*. *lactis* cells without compound is estimated to be 6.3 μM, and it increases to 22 μM upon riboflavin ligation.

Based on the *K*_D_^eff^ of LmrR to the *lmrCD* operator (6.3 μM), the intracellular LmrR concentration (3.2 μM), and the 2:1 binding stoichiometry, the population of the *lmrCD* operator that is not occupied by LmrR was estimated to be 46% under normal conditions (Fig. 4D, black). Therefore, the expression of the *lmrCD* gene is not fully suppressed by LmrR, allowing the basal expression of the *lmrCD* genes. The population of the unoccupied *lmrCD* operator is estimated to increase to 76% upon the ligation of the compound (Fig. 4D, magenta), which leads to a substantial increase (1.7-fold) in the transcription of the *lmrCD* genes. The estimated transcription level agrees well with the previously reported expression profiles of the *lmrCD* genes^11, 12^. Therefore, these results indicate that LmrR performs its sensory functions in the MDR system through a dynamic shift in its position on the genomic DNA, which is coupled to the compound-induced changes in the conformational ensembles.

## Discussion

### LmrR binds to the *lmrCD* operator with a 2:1 stoichiometry

The ITC analyses revealed that LmrR binds to the PadR-consensus sequence of the *lmrCD* operator with an unexpected 2:1 stoichiometry (Fig. 2A). Although the structures of the protein-DNA complexes are not available for either PadR or PadR-like protein family members, it has been suggested that they interact with the consensus DNA sequence with 1:1 stoichiometry^16^. The unexpected 2:1 stoichiometry might be caused by the non-canonical 10 bp separation of the two half-sites in the *lmrCD* operator. Indeed, in the *lmrR* operator site, the two half-sites are separated by the canonical 8 bp sequence, and the binding stoichiometry between the LmrR dimer and the *lmrR* operator site was 1:1 (Supplementary Fig. S5). The 2:1 binding stoichiometry between the LmrR dimer and the *lmrCD* operator may contribute to improving the sensitivity to small affinity changes induced by the binding of promiscuous compounds. If the 1:1 binding stoichiometry is assumed instead of the 2:1 binding stoichiometry, then the basal activity seems to be too high and the expected change in the transcriptional activity upon compound binding becomes smaller (Fig. 4E). Therefore, the 2:1 binding stoichiometry enhances the effect of the affinity reduction, caused by the promiscuous compound ligation, on the release of the transcriptional repression.

The 2:1 binding stoichiometry has also been reported in the interaction between a dimeric multidrug binding transcriptional repressor, QacR, and its inverted-repeat operator site in the *qacA* gene^22^, as well as DtxR-DNA and Ms6564-DNA interactions^23, 24^. QacR belongs to the TetR transcriptional repressor family, but its binding site is unusually long, as compared to those of other TetR family members that exhibit 1:1 binding stoichiometry (28 bp vs 15 bp). However, the cooperativities of the two dimers in the DNA binding are quite different between LmrR and QacR. Biochemical analyses demonstrated the that only the 2:1 QacR dimer:DNA complex, but not the 1:1 complex, was present in solution, suggesting the strong cooperativity between two QacR dimers in DNA binding^22^. QacR binding led to the widening of the major grooves, which may contribute to the cooperative QacR interactions. In contrast, the formation of the 1:1 LmrR dimer-DNA complex together with the 2:1 complex was observed in our study (Supplementary Fig. S2). Therefore, although these two proteins share the same binding stoichiometry, their binding modes to their respective operators are apparently different.

It should also be noted that the DNA footprint analyses indicated that LmrR binds to a wide range of *lmrCD* promoter/operator sequences^11, 12^, including the putative −10 and −35 sites in the *lmrCD* promoter. We confirmed that LmrR also binds to the −10/−35 promoter sequence of *lmrCD*, causing severe broadening of the Ile ^1^H_δ1_-^13^C_δ1_ resonances (Supplementary Fig. S6). However, the addition of the same concentration of the *lmrCD* operator to the preformed LmrR-*lmrCD* promoter complex resulted in a spectrum identical to that of the *lmrCD* operator-bound state Supplementary Fig. S6). Therefore, LmrR can bind to both *lmrCD* promoter/operator sites, but the primary higher-affinity binding site is the PadR-consensus sequence of the *lmrCD* operator.

### The conformational equilibrium of LmrR defines its binding affinity to the *lmrCD* operator

Although the allosteric coupling between the compound and the DNA binding site is assumed to be the structural basis for the compound-induced reduction in the operator binding affinity, the direct correlation between the α4 helix conformational equilibrium and the binding affinity of LmrR for the PadR-consensus sequences has not been investigated. We showed that the conformationally biased mutants with higher a propensity to adopt the *upper* α4 helix conformations significantly reduced the affinity to the *lmrCD* operator (Fig. 2C). In addition, the degree of the affinity reduction correlated with the conformational discrepancy of these mutants against the *lmrCD* operator-bound state of wild-type LmrR (Fig. 2B and C). The results indicated that the change in the α4 helix conformational ensemble and the decrease in the LmrR affinity to the *lmrCD* operator are directly correlated. In our study, riboflavin reduced the LmrR affinity to the *lmrCD* operator by 3.4-fold (Fig. 3D). Given the shared structural and energetic properties in the bound states among the compounds (Fig. 3B and C)^17^, a similar affinity reduction would be expected for the ligations of other compounds to LmrR. These observation further supports that the multidrug recognition and the transcription regulation of LmrR is coupled via a conformational selection mechanism^18^. The transcriptional regulation by conformational selection mechanism has been suggested to bacterial repressors and eukaryotic nuclear receptors^25, 26^, indicating the generality in the molecular mechanisms for ligand induced transcriptional regulations.

### The non-specific DNA adsorption contributes to the *in vivo* transcriptional regulation

Due to the small reduction in the LmrR binding affinity (less than 10-fold) to the *lmrCD* operator upon the compound ligation and the high endogenous concentrations of LmrR in *L*. *lactis* (3.2 μM, Fig. 4A), a DNA dissociation model that assumes a simple equilibrium between the free state and the *lmrCD* operator bound state cannot explain the transcriptional regulation by LmrR (Fig. 4B). In the DNA dissociation model, the estimated population of the *lmrCD* operator that is free of LmrR is always zero.

Given the high concentrations of genomic DNA (3.6 mM) and the non-specific DNA affinity of LmrR with the mid μM *K*_D_ value (Fig. 4C), LmrR is always located on the genomic DNA and thus the contribution of the non-specific DNA adsorption to the transcriptional regulation should be considered as it has been pointed out for repressors that are regulated by specific inducers^21, 27, 28^. The *K*_D_^eff^ value of LmrR to the *lmrCD* operator under the equilibrium between the specific operator-bound and non-specific DNA adsorption states in the genomic DNA was 6.3 μM, which is close to the intracellular concentration of endogenous LmrR (Fig. 4A, 3.2 μM). The concordance of the *K*_D_^eff^ to the *lmrCD* operator and the intracellular concentration in the non-specific DNA adsorption model seems to maximize the effects of the compound-induced affinity reduction on the transcriptional regulation (Fig. 4D), which are further enhanced by the 2:1 stoichiometry, as discussed above (Fig. 4E). It should be noted that LmrR binds to its own operator, and the affinity of LmrR for the *lmrR* operator (Supplementary Fig. S6, 110 nM) is similar to that for the *lmrCD* operator (Fig. 2A, 64 nM). Thus, the autorepression mechanism seems to ensure that the intracellular concentration of LmrR is autonomously adjusted to match the *K*_D_^eff^ of LmrR to the *lmrCD* operator.

A report indicated that the intracellular concentration of prokaryotic transcriptional repressors is on average 10 times higher than that of transcriptional activators, and the ligand-dependent transcription factors are present at higher intracellular concentrations, as composed to the ligand-independent transcription factors^29^. The reported median copy number of ligand-dependent transcription factors is ~600, which corresponds to 0.9 μM in *L*. *lactis* cells. Therefore, the estimated endogenous concentration of LmrR (3.2 μM) seems to be reasonable as a ligand-dependent transcription factor. The high concentration of transcription factors has been considered to allow their rapid localization to specific DNA sites in general^30^; thus, the relatively high endogenous concentration of LmrR would contribute to the rapid transcriptional response against toxic compounds.

As discussed in above, the reduction of the affinity of LmrR to the *lmrCD* operator caused by promiscuous compound ligations is relatively small, as compared to the affinity reduction of other repressors with specific inducers. Evidences support that non-specific DNA adsorption contributes to the transcriptional regulation of the *lac* repressor as well^21, 27^; however, the intracellular concentration of the *lac* repressor is not autonomously regulated by the autorepression. The *lac* repressor is not encoded within the same operon as the *lacZ*, *lacY*, *and lacA* genes, which are controlled by the *lac* repressor, and there is no *lac* repressor binding site in the promoter/operator region of its own gene. This might be due to the fact that strict control of the intracellular concentration is not necessary for the *lac* repressor, because a significant decrease in the binding affinity can easily overcome any concentration mismatch.

In contrast, the autorepression mechanism seems to be rather common among the multidrug transcription repressors. MexR, a member of the MarR family, is a multidrug binding transcriptional regulator of the MexAB-OprM transporter^31^. In the MDR system, *mexR*, *mexAB*, and *oprM* form a gene cluster, and MexR auto-regulates its transcription^32^. A similar autorepression mechanism was suggested for the *acrR* and *acrA* genes and for other genes as well^6^. Therefore, MDR systems appear to have elaborate mechanisms for efficient transcriptional regulation, through minimal changes in the operator affinity that are induced by the promiscuous compound ligations. As a consequence, the non-specific DNA adsorption and the autonomous regulation of their concentrations synergistically contribute to the functional outcomes (Fig. 4D).

### The non-specific DNA adsorption model explains the basal and induced expression of the *lmrCD* genes *in vivo*

The constitutive basal expression of the *lmrCD* genes in *L*. *lactis* is reportedly important for the basic-level of resistance to the toxic compounds^11, 12^. The knockout of the *lmrCD* genes leads to the hypersensitivity of *L*. *lactis* to toxic compounds^10^. Upon exposure to toxic compounds, the expression of the *lmrCD* genes is increased by up to two-fold^11, 12^. The non-specific DNA adsorption model with the 2:1 binding stoichiometry allows a significant basal expression level, in agreement with the predicted enhancement of the *lmrCD* gene transcription (1.7-fold, Fig. 4D and E). Therefore, the non-specific DNA adsorption model represents the molecular basis for the *in vivo* transcriptional regulation of the *lmrCD* genes by LmrR^11, 12^.

## Conclusion

Here, we propose the transcriptional regulation mechanism of the multidrug binding transcriptional repressor, LmrR, through the dynamic balance and relocation between the specific operator interaction and the non-specific DNA adsorption states in the genomic DNA. In this mechanism, the autonomous regulation of its own gene seems to be important to maintain the optimal endogenous concentration of LmrR, to express the maximal degree of changes in the transcription repression upon compound ligations. The transcription regulation in a single gene cluster also seems to be quite reasonable to achieve the maximal utilization of the limited genomic resources of prokaryotes. The 2:1 binding stoichiometry between the LmrR dimer and the *lmrCD* operator further enhances the sensitive regulation upon compound ligation, while allowing the constitutive basal expression of the *lmrCD* genes for maintaining the minimal resistance to toxic compounds. Therefore, LmrR represents a dynamic type of transcriptional regulation in a prokaryotic multidrug resistance system, in which the promiscuous compound ligations are coupled to the positioning of the transcriptional repressor on genomic DNA via the equilibrium between operator-bound and non-specific DNA-adsorption states, to play a significant role in determining the degree of transcriptional repression.

## Materials and Methods

All chemicals were purchased from WAKO or Sigma, unless otherwise stated. All stable isotope-labeled materials were acquired from Cambridge Isotope Laboratories. The polyclonal antibody against LmrR was developed by MBL Life Science, using recombinant LmrR as the antigen. *E*. *coli* genomic DNA was purchased from Affymetrix, dissolved in buffer containing 10 mM NaPi (pH 6.8) and 100 mM NaCl, and sonicated for 5 min to obtain smaller fragments with an average size of ~500 bp.

### DNA oligo sequences

Oligonucleotide sequences used in this study are as follows:

PadR-consensus sequence in the *lmrCD* operator (33 bp, bold and underlined: PadR-consensus motif): 5′-CAATTTA**ATGT**AAAGTAGTTT**ACAT**TATTTAAC-3′

The −10/−35 sequence of the *lmrCD* promoter (35 bp, bold and underlined: −10/−35 motifs): 5′-GC**TTGTTT**ACTAAAAAAAATAATGT**TATAAT**TATC-3′

PadR-consensus sequence of the *lmrR* operator (33 bp, bold and underline: PadR-consensus motif): 5′-TACATAGTA**ATGT**GAAGTATA**ATAT**ACTTTGTT-3′.

### Preparation of LmrR

The sequence encoding C-terminal His_6_-tagged LmrR was cloned into the pET28b vector (Novagen), as previously described^17^. LmrR mutants were constructed using the QuikChange^TM^ strategy (Agilent Technology). Expression and purification were performed as previously described^17^. For selective ^13^CH_3_-labeling of the Ile (Ile-δ1), Leu, and Val methyl groups, the growth medium was supplemented with 100 mg/L of [methyl-^13^C, 3,3-^2^H_2_]-α-ketobutyric acid and 100 mg/L of [3-methyl-^13^C, 3,4,4,4-^2^H_4_]-α-ketoisovaleric acid, 30 min prior to the addition of IPTG. Purified proteins were flush frozen in liquid N_2_ and stored at −80 °C until further use.

### ITC measurements

Calorimetric titrations were performed using a VP-ITC microcalorimeter (MicroCal) at 25 °C, with the same buffer used in NMR experiments. Protein samples were extensively dialyzed against ITC/NMR buffer, containing 10 mM NaPi (pH 6.8) and 100 mM NaCl, before the experiments. The sample cell was filled with 5–10 µM LmrR dimer, and the injection syringe contained 50–100 µM of the oligo DNA or 50 μM of riboflavin. LmrR and *lmrCD* oligo were quantified by UV absorbance values of 280 nm (ε = 39,800 as dimer) and 260 nm (ε = 227,000), respectively. After a preliminary 3 µL injection, 24 subsequent 10 µL injections were performed. In the opposite titration experiment, the sample cell was filled with 2 µM DNA, and the injection syringe typically contained 50 µM of LmrR dimer. For riboflavin titrations, 5% of DMSO was added to the buffer, in order to increase the solubility of the compound. The data were fitted using the one-site binding model embedded in Origin 7.0 (MicroCal).

### NMR experiments

All experiments were performed using either Bruker Avance-600 MHz or Avance III-800 MHz spectrometers equipped with cryogenic triple resonance probes. All spectra were recorded using 10 mM NaPi buffer (pH 6.8) containing 100 mM NaCl, in either 90% H_2_O/10% D_2_O or 100% D_2_O, depending on the experiments. The typical concentration of LmrR was 0.1–0.2 mM as a monomer. Unless otherwise stated, the experiments were performed at 298 K. Spectra were processed using TOPSPIN (Bruker Biospin) and analyzed with Sparky. The assignments of the Ile, Leu, and Val methyl resonances of LmrR were established previously^17^.

The rotameric equilibria of the Ile χ^2^ angles were deduced from the Ile (δ1) methyl ^13^C chemical shifts. The ^13^C chemical shifts of methyl signals are reportedly dependent on the sidechain rotamer, as demonstrated by theoretical and experimental analyses^20^. The population in the *trans* rotameric state (*p*_t_) for each residue was calculated according to the absolute chemical shift values of the methyl ^13^C signals (*δ*_obs_; ppm), using the equation (2):

2 $${}^{13}{\rm{C}}_{{\rm{\delta }}1}\mathrm{Ile}:{\delta }_{{\rm{obs}}}=9.3+5.5{{\rm{p}}}_{{\rm{t}}}$$

If the equation yielded a *p*_t_ value >1 or <0, then *p*_t_ was fixed to 1 (all *trans*) or 0 (all *gauche−*), respectively^20^.

### Quantification of the cellular concentration of LmrR

The concentration of endogenous LmrR in *L*. *lactis* cells was estimated by western blot analyses, using an polyclonal antibody against LmrR. *L*. *lactis* cells were grown overnight at 30 °C in M17 media supplemented with 0.5% lactose. Cells were collected by centrifugation (10,000 *g*, 30 min), lysed by sonication, and further digested by Cryonase nuclease (RiboSolutions) at 4 °C for 1 hr. The difference between the wet volume of cells after centrifugation and the dry volume after overnight lyophilization was used as the total volume of cytosol, which is typically 80% of the wet volume (assuming 1 g equal 1 ml). Western blotting was performed according to the standard protocol, using an iBlot dry blotting system (Invitrogen). The image was obtained by the ImageQuant LAS4000 system (GE healthcare) and the quantification was performed with the ImageQuant TL program (GE healthcare), using the purified recombinant LmrR as the calibration standard.

## Electronic supplementary material

Supplementary Figures

## References

[1] Ma D, Cook DN, Hearst JE, Nikaido H (1994). Efflux pumps and drug resistance in Gram-negative bacteria. Trends in Microb.

[2] Saier MH (1998). Evolutionary origins of multidrug and drug-specific efflux pumps in bacteria. Faseb j.

[3] van Veen HW, Konings WN (1998). Structure and function of multidrug transporters. Adv Exp Med Biol.

[4] Geick A, Eichelbaum M, Burk O (2001). Nuclear receptor response elements mediate induction of intestinal MDR1 by rifampin. J Biol Chem.

[5] Riordan JR (1985). Amplification of P-glycoprotein genes in multidrug-resistant mammalian cell lines. Nature.

[6] Grkovic S, Brown MH, Skurray RA (2002). Regulation of Bacterial Drug Export Systems. Microb Mol Biol Rev.

[7] Schumacher MA, Brennan RG (2002). Structural mechanisms of multidrug recognition and regulation by bacterial multidrug transcription factors. Mol Microbiol.

[8] Bolhuis H (1994). Proton motive force-driven and ATP-dependent drug extrusion systems in multidrug-resistant Lactococcus lactis. J Bacteriol.

[9] Lubelski J, Mazurkiewicz P, van Merkerk R, Konings WN, Driessen AJ (2004). ydaG and ydbA of Lactococcus lactis encode a heterodimeric ATP-binding cassette-type multidrug transporter. J Biol Chem.

[10] Lubelski J (2006). LmrCD is a major multidrug resistance transporter in Lactococcus lactis. Mol Microbiol.

[11] Agustiandari H, Peeters E, de Wit JG, Charlier D, Driessen AJ (2011). LmrR-mediated gene regulation of multidrug resistance in Lactococcus lactis. Microbiology.

[12] Agustiandari H, Lubelski J, van den Berg van Saparoea HB, Kuipers OP, Driessen AJ (2008). LmrR is a transcriptional repressor of expression of the multidrug ABC transporter LmrCD in Lactococcus lactis. J Bacteriol.

[13] Wade H (2010). MD recognition by MDR gene regulators. Curr Opin Structl Biol.

[14] Gury J, Barthelmebs L, Tran NP, Diviès C, Cavin J-F (2004). Cloning, Deletion, and Characterization of PadR, the Transcriptional Repressor of the Phenolic Acid Decarboxylase-Encoding padA Gene of Lactobacillus plantarum. Appl Env Microb.

[15] Madoori PK, Agustiandari H, Driessen AJ, Thunnissen AM (2009). Structure of the transcriptional regulator LmrR and its mechanism of multidrug recognition. Embo j.

[16] van der Berg JP, Madoori PK, Komarudin AG, Thunnissen AM, Driessen AJ (2015). Binding of the Lactococcal Drug Dependent Transcriptional Regulator LmrR to Its Ligands and Responsive Promoter Regions. PLoS One.

[17] Takeuchi K, Tokunaga Y, Imai M, Takahashi H, Shimada I (2014). Dynamic multidrug recognition by multidrug transcriptional repressor LmrR. Sci Rep.

[18] Monod J, Wyman J, Changeux J-P (1965). On the nature of allosteric transitions: A plausible model. Journal of Molecular Biology.

[19] Barkley MD, Riggs AD, Jobe A, Bourgeois S (1975). Interaction of effecting ligands with lac repressor and repressor-operator complex. Biochemistry.

[20] Hansen DF, Neudecker P, Kay LE (2010). Determination of isoleucine side-chain conformations in ground and excited states of proteins from chemical shifts. J Am Chem Soc.

[21] Lin S-y, Riggs AD (1975). The general affinity of lac repressor for *E. coli* DNA: Implications for gene regulation in procaryotes and eucaryotes. Cell.

[22] Schumacher MA (2002). Structural basis for cooperative DNA binding by two dimers of the multidrug-binding protein QacR. EMBO J.

[23] White A, Ding X, vanderSpek JC, Murphy JR, Ringe D (1998). Structure of the metal-ion-activated diphtheria toxin repressor/tox operator complex. Nature.

[24] Yang S (2013). Structural Basis for Interaction between Mycobacterium smegmatis Ms6564, a TetR Family Master Regulator, and Its Target DNA. The Journal of Biological Chemistry.

[25] Changeux J-P, Edelstein S (2011). Conformational selection or induced fit? 50 years of debate resolved. F1000 Biology Reports.

[26] Liguori A (2016). Molecular Basis of Ligand-Dependent Regulation of NadR, the Transcriptional Repressor of Meningococcal Virulence Factor NadA. PLoS Pathog.

[27] Hippel PHV, Revzin A, Gross CA, Wang AC (1974). Non-specific DNA Binding of Genome Regulating Proteins as a Biological Control Mechanism: 1. The lac Operon: Equilibrium Aspects. Proceedings of the National Academy of Sciences.

[28] Gerland U, Moroz JD, Hwa T (2002). Physical constraints and functional characteristics of transcription factor–DNA interaction. Proceedings of the National Academy of Sciences.

[29] Li GW, Burkhardt D, Gross C, Weissman JS (2014). Quantifying absolute protein synthesis rates reveals principles underlying allocation of cellular resources. Cell.

[30] von Hippel PH (2007). From “simple” DNA-protein interactions to the macromolecular machines of gene expression. Annu Rev Biophys Biomol Struct.

[31] Poole K (1996). Expression of the multidrug resistance operon mexA-mexB-oprM in Pseudomonas aeruginosa: mexR encodes a regulator of operon expression. Antimicrobl Agen Chemoth.

[32] Evans K, Adewoye L, Poole K (2001). MexR Repressor of the mexAB-oprMMultidrug Efflux Operon of Pseudomonas aeruginosa: Identification of MexR Binding Sites in the mexA-mexRIntergenic Region. J Bacteriol.

