# Supplementary material for: Dynamic equilibrium on DNA defines transcriptional regulation of a multidrug binding transcriptional repressor, LmrR
Source: Sci Rep. 2017 Mar 21;7:267. doi: 10.1038/s41598-017-00257-x (PMC5428041; doi:10.1038/s41598-017-00257-x)
Supplement: Supplementary file 1 — Supplementary Figures [file 41598_2017_257_MOESM1_ESM.pdf]

Supplemental figures for

Dynamic equilibrium on DNA defines transcriptional regulation of a multidrug binding transcriptional repressor, LmrR

Authors:

Koh Takeuchi[1,2], Misaki Imai[3], and Ichio Shimada[1,4]*

Affiliations:

[1]Biomedicinal Information Research Center & Molecular Profiling Research Center for Drug Discovery, National Institute of Advanced Industrial Science and Technology, Aomi 2-3-26, Koto-ku, Tokyo 135-0064, Japan. [2]PRESTO, JST, Aomi 2-3-26, Koto-ku, Tokyo 135-0064, Japan.[3]Research and Development Department, Japan Biological Informatics Consortium, Aomi 2-3-26, Koto-ku, Tokyo 135-0064, Japan. [4]Graduate School of Pharmaceutical Sciences, The University of Tokyo, 7-3-1 Hongo, Bunkyo-ku, Tokyo 113-0033, Japan.

***Correspondence to:**

Prof. Ichio Shimada, Ph.D, Graduate School of Pharmaceutical Sciences, The University of Tokyo, Hongo 7-3-1, Bunkyo-ku, Tokyo 113-0033, Japan. Phone & Fax: +81-3-3815-6540

Email: shimada@iw-nmr.f.u-tokyo.ac.jp

Supplemental Figures:

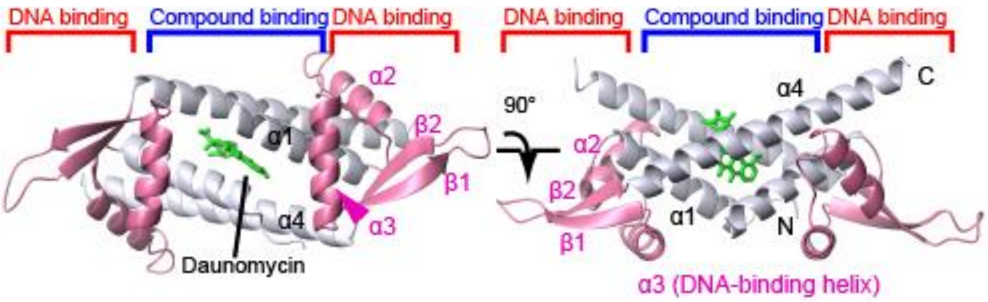

Figure S1: Ribbon diagrams of LmrR in complex with daunomycin. The compound (green sticks) binds to the pore at the dimeric center, which are formed by α1 and α4 helices. The α3 helix is considered to directly interact to the major groove of DNA.

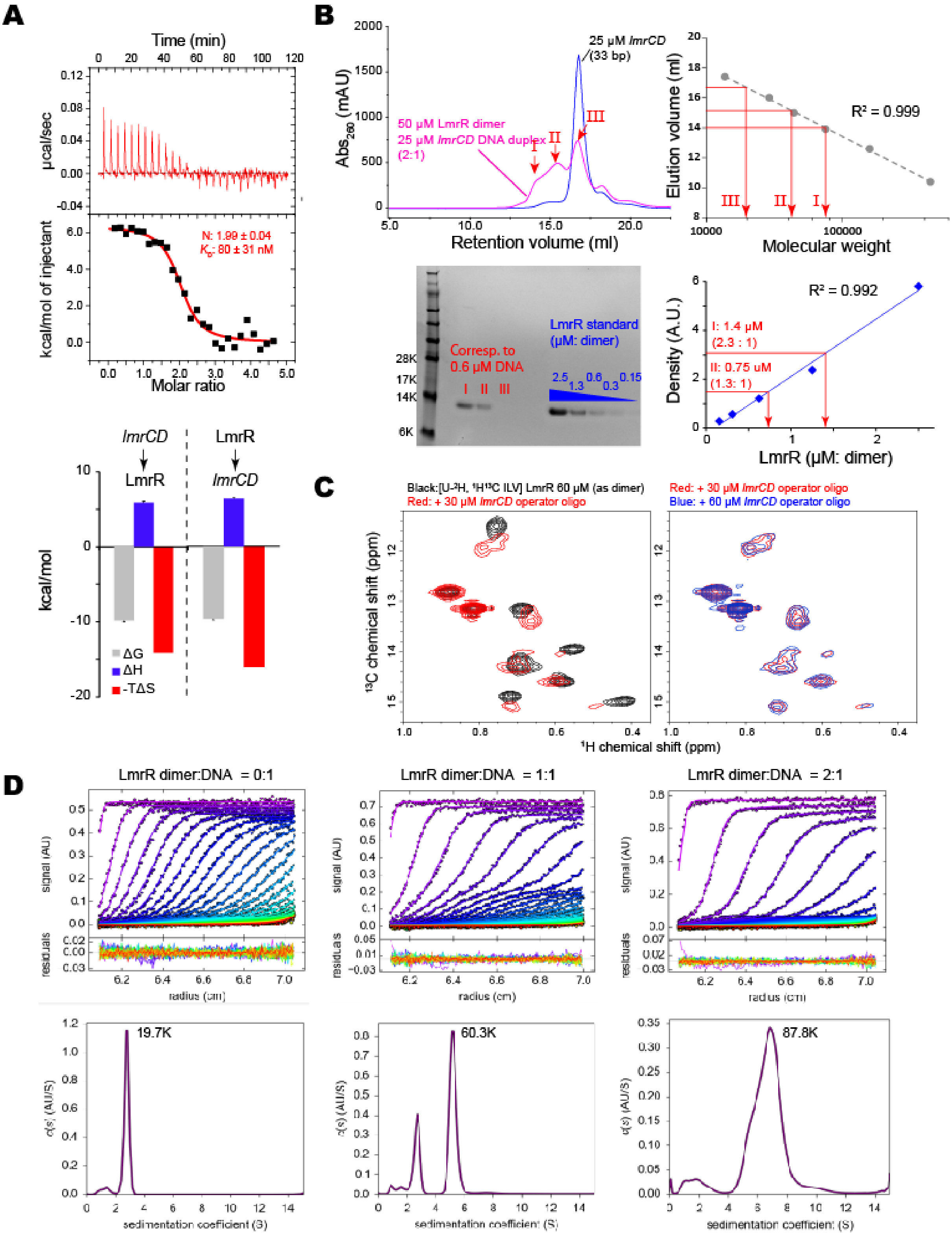

Figure S2: Determination of the binding stoichiometry of the interaction between LmrR and the PadR-consensus sequence in the *lmrCD* operator. (A) ITC measurements of the interaction between the *lmrCD* operator and LmrR. LmrR was titrated against the *lmrCD* operator. The thermodynamic nature of the interaction between LmrR and the *lmrCD*

operator is shown (left: the *lmrCD* operator titration against LmrR, right: LmrR titration against the *lmrCD* operator). (B) SEC analysis of the LmrR-*lmrCD* operator complex. Top left: The *lmrCD* operator alone (blue) or the *lmrCD* operator in complex with LmrR (magenta, 2:1 LmrR dimer:DNA complex) was applied to a Superdex 200 10/300 column (GE Healthcare), equilibrated with 10 mM sodium phosphate buffer (NaPi, pH 6.8) containing 100 mM NaCl. The mixture were diluted in the column and the estimated maximum concentrations of DNA and LmrR in the column is ~2 μM and ~4 μM, respectively. Top right: Estimation of apparent molecular weight from the elution volume. Fractions I, II, and III have apparent molecular weight of 75K, 41K, and 20K, which are close proximity to the theoretical values, 2:1 LmrR dimer: *lmrCD* operator complex (80.8 K), 1:1 LmrR dimer: *lmrCD* operator complex (50.4 K), and *lmrCD* operator DNA (20K), respectively. Bottom: Determination of the binding stoichiometry of the LmrR-*lmrCD* operator complex. Fractions I and II from the SEC analyses were used for SDS-PAGE at the concentrations corresponding to 0.6 μM DNA (left), and the protein content in the fraction was estimated from the calibration curve of the purified LmrR. Fractions I and II contain 1.4 μM and 0.75 μM LmrR dimer, respectively, indicating the formation of the 2:1 and 1:1 LmrR dimer: *lmrCD* operator complexes in solution (right). The presence of both the 2:1 and 1:1 LmrR dimer: *lmrCD* operator complexes is reasonable for the dilution and separation effects in the SEC analysis. It also indicates that strong cooperativity between the LmrR dimer in their DNA binding would not be expected. Data are representative of two independent experiments. (C) Titration of *lmrCD* operator to [U-^{2}H, ^{1}H^{13}C-ILV] LmrR. The addition of 30 μM of the *lmrCD* operator oligo to 60 μM of LmrR induces the chemical shift changes to the fully bound conformation (left) and addition of the extra *lmrCD* operator did not induced further chemical shift changes (right). (D) Analytical ultracentrifugation experiments. The sedimentation profiles (top) and the sedimentation coefficient profile (bottom) of

sedimentation velocity experiments for indicated conditions are shown. Sedimentation velocity experiments were conducted with an Optima XLI (Beckman-Coulter, Fullerton, CA), using a 4-hole An60Ti rotor at 20°C. LmrR and lmrCD operator were dissolved in the same buffer as NMR experiments (10 mM Sodium Phosphate (pH6.8), 100 mM NaCl). The concentration profiles were monitored by absorption at 280 nm with a 3-mm path length centerpieces. Sedimentation velocity data were acquired at rotor speeds of 50,000 rpm with no time intervals. The partial specific volume of DNA was set to 0.55 and that of the protein was calculated to be 0.724 cm^3/g based of the amino acid sequence using SEDNTERP software[1]. The partial specific volumes, v_{bar}, for the protein-DNA complexes were calculated to be 0.654 and 0.681 for 1:1 and 2:1 complex, respectively, assuming that the v_{bar} is the weight average of the protein and DNA. The buffer densities and viscosities were calculated from the composition also using SEDNTERP. Sedimentation velocity data were analyzed by SEDFIT assuming that the frictional ratio is common to all the molecular species[2]. The concentration of DNA in the sample was fixed to 8 μM, whereas the concentration of LmrR dimer were varied to be 0 μM, 8 μM, and 16 μM (0:1, 1:1, and 2:1 stoichiometry of LmrR dimer:DNA, respectively). DNA only sample showed the peak sedimentation value of 2.84S with the estimated molecular weight of 19.7 K that corresponds to *lmrCD* operator (33 mer, 20 K). The 1:1 and 2:1 samples showed the peak sedimentation values of 5.37S and 7.26S and the estimated molecular weights were 60.3 K and 87.8K, respectively. These are closely matched with the molecular weight of 1:1 and 2:1 LmrR dimer-DNA complexes (50.4 K and 80.8 K). The 1:1 and 2:1 samples also showed the sedimentation distribution corresponding to free DNA and 1:1 complex, respectively. The sedimentation distribution peak of 1:1 and 2:1 complexes in the 2:1 sample was not fully separated suggesting that the two molecular species are in dynamic equilibrium.

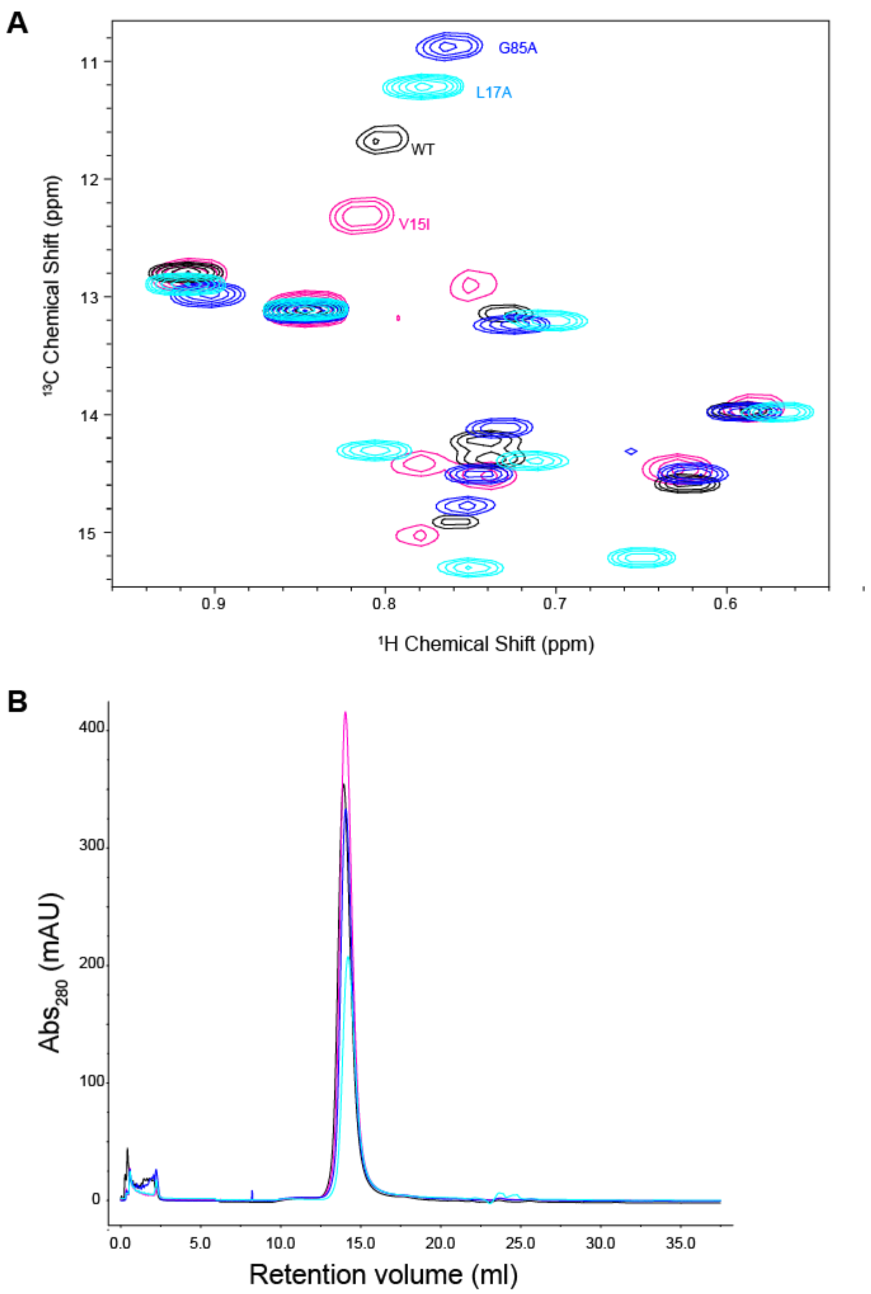

Figure S3: Characterization of conformational biased mutants. (A) $^{1}H^{13}C$ HMQC spectra of WT and V15I, L17A, G85A mutants. Each spectrum was color coded in the same way as in Figure 2. The similar signal dispersion pattern of the mutants indicates that there is no significant structural distortion. (B) SEC analysis of WT and V15I, L17A, G85A mutants. Each elution profile was color coded in the same way as in Figure 2. The identical retention times indicate there is no monomerization and aggregation of LmrR caused by the mutations. Superdex75 10/300 column was used for the analysis. Elution buffer was 10 mM phosphate buffer (pH 6.8) NaCl 300 mM. Data are representative of two independent experiments.

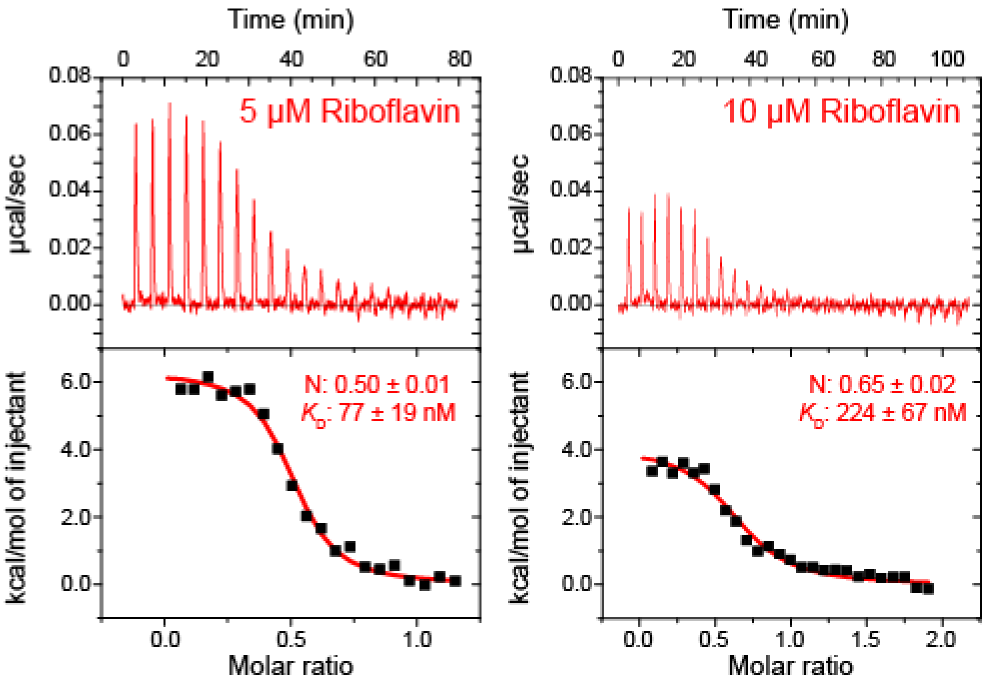

Figure S4: Reduction of LmrR binding affinity to the *lmrCD* operator in the presence of riboflavin. The *lmrCD* operator was titrated against LmrR in the presence of the indicated concentrations of riboflavin. Data are representative of two independent experiments.

lmrR operator: TACATAGTA**ATGT**GAAGTATA**ATAT**ACTTTGTT

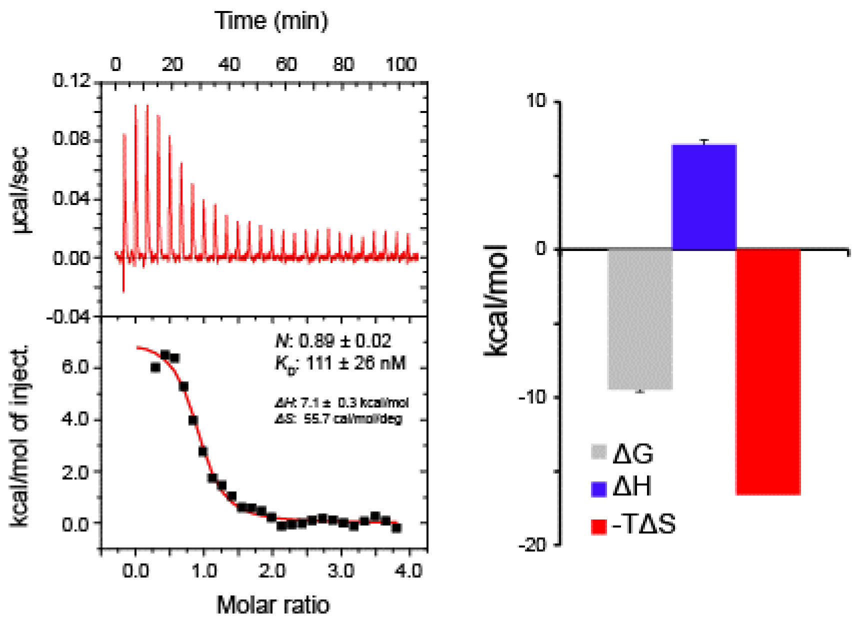

Figure S5: Binding of LmrR to the *lmrR* operator. The *lmrR* operator was titrated against LmrR. The sequence of the *lmrR* operator is also shown. Data are representative of two independent experiments.

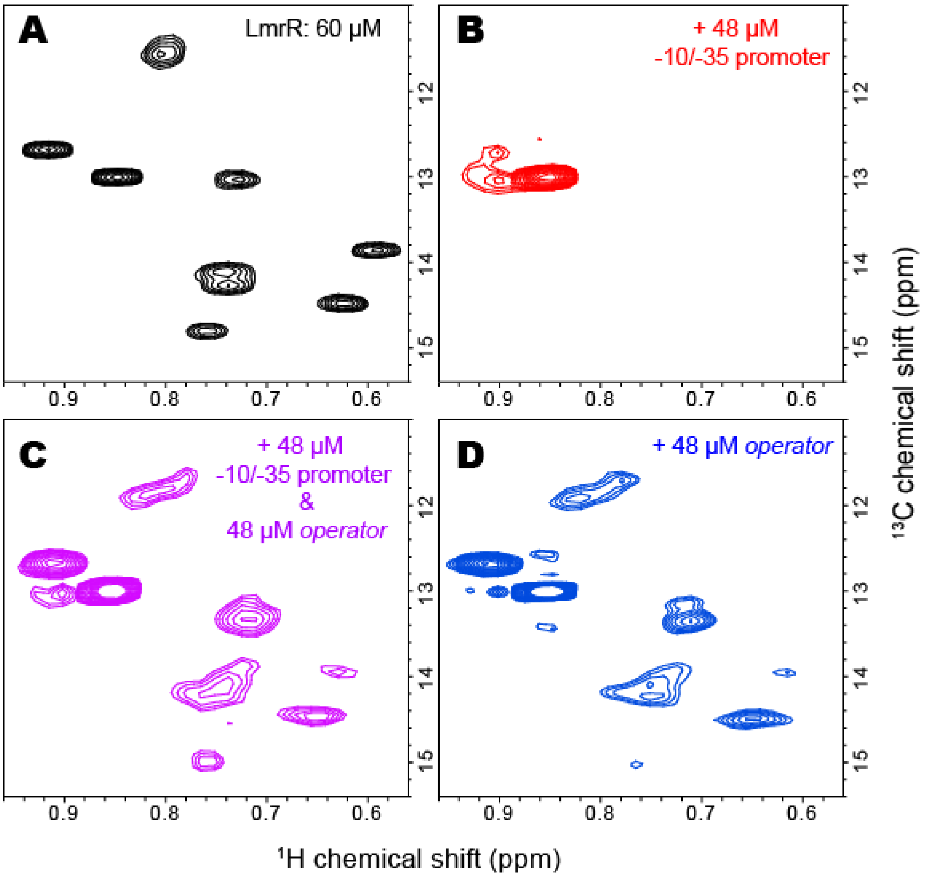

Figure S6: The PadR-consensus operator sequence out-competes the -10/-35 *lmrCD* promoter sequence. 2D ^{1}H-^{13}C HMQC spectra of (A) 30 µM LmrR dimer, (B) 30 µM LmrR dimer in a complex with 48 µM -10/-35 promoter, (C) the addition of 48 µM PadR-consensus operator to (B), and (D) 30 µM LmrR dimer in a complex with 48 µM PadR-consensus operator. The identical spectra of (C) and (D) indicate that the PadR-consensus operator out-competes the -10/-35 *lmrCD* promoter for LmrR binding. Data are representative of two independent experiments.

Supplemental references

1. Laue, T., Shah, B., Ridgeway, T. & Pelletier, S. Computer-aided interpretation of analytical sedimentation data for proteins. *Analytical ultracentrifugation in biochemistry and polymer science*, 90–125 (1992).
2. Schuck, P. Size-distribution analysis of macromolecules by sedimentation velocity ultracentrifugation and lamm equation modeling. *Biophys J* **78**, 1606-19 (2000).
